# The Stability of Medicinal Plant microRNAs in the Herb Preparation Process

**DOI:** 10.3390/molecules23040919

**Published:** 2018-04-16

**Authors:** Wenyan Xie, Matthias F. Melzig

**Affiliations:** Institute of Pharmacy, Freie Universitaet Berlin, Koenigin-Luise-Str. 2+4, 14195 Berlin, Germany; matthias.melzig@fu-berlin.de

**Keywords:** microRNAs, medicinal plants, stability, herb preparations, *Viscum album* L., mistletoe

## Abstract

Herbal medicine is now globally accepted as a valid alternative system of pharmaceutical therapies. Various studies around the world have been initiated to develop scientific evidence-based herbal therapies. Recently, the therapeutic potential of medicinal plant derived miRNAs has attracted great attraction. MicroRNAs have been indicated as new bioactive ingredients in medicinal plants. However, the stability of miRNAs during the herbal preparation process and their bioavailability in humans remain unclear. *Viscum album* L. (European mistletoe) has been widely used in folk medicine for the treatment of cancer and cardiovascular diseases. Our previous study has indicated the therapeutic potential of mistletoe miRNAs by using bioinformatics tools. To evaluate the stability of these miRNAs, various mistletoe extracts that mimic the clinical medicinal use as well as traditional folk medicinal use were prepared. The mistletoe miRNAs including miR166a-3p, miR159a, miR831-5p, val-miR218 and val-miR11 were quantified by stem-loop qRT-PCR. As a result, miRNAs were detectable in the majority of the extracts, indicating that consumption of medicinal plant preparations might introduce miRNAs into mammals. The factors that might cause miRNA degradation include ultrasonic treatment, extreme heat, especially RNase treatment, while to be associated with plant molecules (e.g., proteins, exosomes) might be an efficient way to protect miRNAs against degradation. Our study confirmed the stability of plant derived miRNAs during herb preparations, suggesting the possibility of functionally intact medicinal plant miRNAs in mammals.

## 1. Introduction

Medicinal preparations derived from natural sources, especially from plants, have been in widespread use since ancient times. Herbal medicine is globally accepted as a valid alternative system of therapy in the form of pharmaceuticals or functional foods. Although ancient medical treatises have documented a large number of medicinal plants, their bioactive constituents and corresponding interactions with human have not been comprehensively characterized. New plant bioactive molecules are being discovered. MicroRNAs (miRNAs) are a class of approximately 22 nucleotides single-stranded non-coding RNA molecules that play crucial roles in gene expression. In 2012, Zhang et al. suggested that the rice miRNAs might enter the mammalian bloodstream and play a functional role in human metabolism [1], providing the first clue that plant miRNAs might impact human health and diseases. Several follow-up studies have supported this finding. MIR2911, which was highly stable in the honeysuckle (*Lonicera japonica* Thunb.) decoction, could be able to enter the lungs of the animals that fed with honeysuckle decoction, where it could directly target various influenza A viruses, suppress their replication process, and protect the mice from influenza infections [2]. Plant miR159, derived from raw or cooked vegetables (e.g., broccoli, soybean), was more abundant in healthy donors in comparison to breast cancer patients [3]. By targeting 3′UTR of transcription factor 7, miR159 significantly suppressed breast cancer cell growth in vitro and in vivo [3]. These studies suggested that plant miRNAs might be absorbed by mammals and regulate mammalian gene expression. We assume that, in medicinal plants, miRNAs might act as the hidden bioactive ingredients that are involved in the therapeutic effects of medicinal plants [4].

One crucial concern regarding functional plant miRNAs is their stability in the preparation process. A commonly held belief is that medicinal plants undergo extensive preparations such as grinding and boiling process, allowing the release of cellular RNase or the generation of extreme heat, which might cause the enzymatic breakdown or thermal degradation of miRNAs. Indeed, the majority of miRNAs were degraded in medicinal plants (e.g., honeysuckle) decoction after boiling for 30 min [2]. However, some dietary plant derived miRNAs (e.g., miR166, miR167, miR168 and miR159) were reported to be degradation resistant, as evidenced by storage, cooking, extremes of pH (e.g., simulated gastric fluid with pH of 1.2) and frequent bowel movement did not abolish miRNAs presented in dietary plants such as rice, soybean, potato, broccoli and cabbage [1,3,5]. Medicinal plants usually undergo various intensive treatments including grinding, drying, soaking, boiling and mechanical treatment. Whether herbal miRNAs could survive these processes, and to what extent miRNAs might be degraded during these treatments, remain to be elucidated.

*Viscum album* L. (*V. album*, Salantaceae, European mistletoe) has been widely used for the treatment of cancer and cardiovascular diseases in Europe. Our previous study indicated that *V. album* miRNAs might be able to regulate some human diseases, especially cancer, cardiovascular diseases and neurological disorders, acting as one of the potential bioactive ingredients in *V. album* [6]. It is essential to determine the stability of mistletoe miRNAs during the herb preparation process.

In this study, we prepared different mistletoe extracts according to the clinical medicinal use as well as the folk medicinal use of mistletoe. The RNAs were isolated and miRNAs were quantified from these extracts. We found that, although the RNA lost its integrity during the whole herb preparation process, some miRNAs survived grinding, drying, storage, and soaking procedures. Being exposed to RNase and ultrasonic treatment might be the major causes for miRNA degradation, while being associated with plant derived macromolecules such as proteins and nanoparticles might protect plant miRNAs during the herb preparation process.

## 2. Results

### 2.1. Total RNA Isolation from Raw Herb Materials and Herb Preparations

The yields and purity of total RNA obtained from mistletoe plants and mistletoe extracts were shown in Table 1. The fresh plant generated high yield of total RNA with 630 μg/g. Interestingly, RNA also presented in the lyophilized extract made from fresh mistletoe plant with the yield of 1404 μg/g. A slightly decreased yield of RNA (1236 μg/g) was found after mechanical treatment. As one gram of lyophilized extract was roughly produced from 5 g of mistletoe plant, which indicated that the fresh mistletoe plant released at least approximate 39–45% of RNA molecules into the water in which the plant was processed.

The total RNA isolated from prepackaged dried mistletoe plant revealed yield of 398 μg/g. After being soaked in the hot water for 10 min, 47% of RNA (1 g of lyophilized extract was roughly produced from 5 g of raw plant materials) was dissolved into the water with an average amount of 931 μg/g. In agreement with previous studies [2], very low amount of RNA (27 μg/g) was obtained from the extract boiled for 30 min, indicating the severe degradation of RNA after 30 min boiling.

The RNA purity was evaluated spectrophotometrically at the absorbance 230, 260 and 280 nm, either low A260/A280 (<1.8) or low A260/A230 (<2.0) value indicated contamination. As shown in Table 2, the RNA isolated from fresh plant, dried plant and various mistletoe preparations showed A260/A280 values around 2.0, and A260/A280 values ranging from 2.2 to 2.5, suggesting the high purity of RNA. However, the total RNA isolated from boiled extract had a low A260/A280 ratio of 1.67 and a low A260/A230 value of 0.8. Proteinase K (Thermo Scientific, Waltham, MA, USA) treatment did not increase these ratios (data not shown), while no conspicuous absorption was observed at 270 nm (data not shown), indicating, rather than protein or phenol contamination, that these low A260/A280 and A260/A230 values might be caused by the much lower concentration of total RNAs in this extract [7].

**Figure 1:**
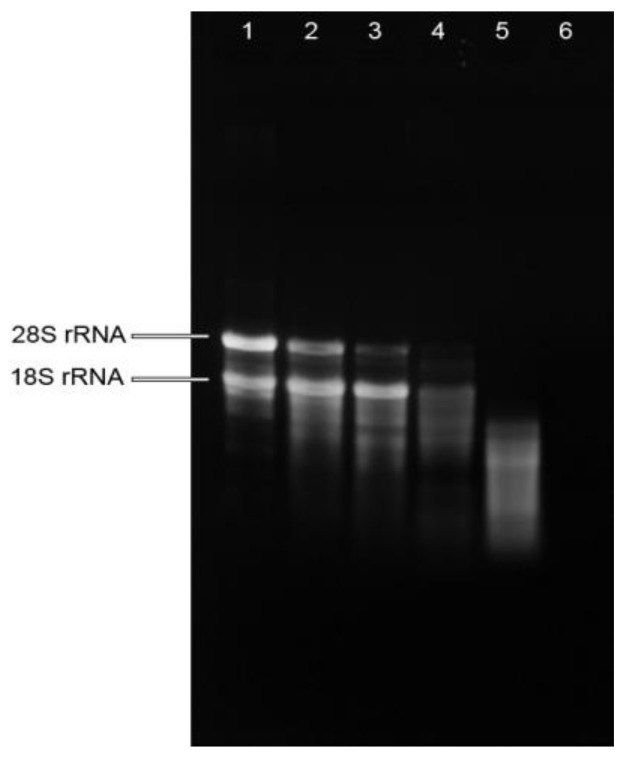
Quality assessment of total RNA isolated from mistletoe preparations by agarose gel electrophoresis. Lane 1: RNA isolated from fresh plant (FP); Lane 2: RNA isolated from mistletoe extract made from FP; Lane 3: RNA isolated from mistletoe extract made from FP treated with mechanical treatment; Lane 4: RNA isolated from dried mistletoe plant (DP); Lane 5: RNA isolated from mistletoe extract made by soaking DP in 80 °C water for 10 min; Lane 6: RNA isolated from mistletoe extract made by boiling DP for 30 min.

RNA integrity was determined by electrophoresis in 1.2% (*w*/*v*) agarose gel. The RNA from fresh plant showed sharp, clear 28S and 18S rRNA bands, and the 28S rRNA band was approximately twice as intense as the 18S rRNA band (Figure 1), indicating the intactness of the RNA from fresh plant. While the intensity of 18S rRNA was stronger than that of 28S rRNA in the RNA isolated from mistletoe extracts made from fresh plants, indicating the degradation of total RNA in these extracts. The RNA isolated from dried plants showed weak rRNA bands and a smeared appearance, suggesting degradation of RNA in dried mistletoe plants. Highly smeared RNA was observed in the mistletoe extract made by soaking dried plant at 80 °C, while bands were barely observed in the RNA isolated from boiling extract, suggesting the degradation of RNA during herb preparation procedures. These results suggested that raw mistletoe plants did release RNA molecules into the water in which the plant was processed, but the enzymatic environment, mechanical treatment as well as heat treatment might encourage the breakdown of total RNA.

### 2.2. The Stability of miRNA during the Herb Preparation Process

**Figure 2:**
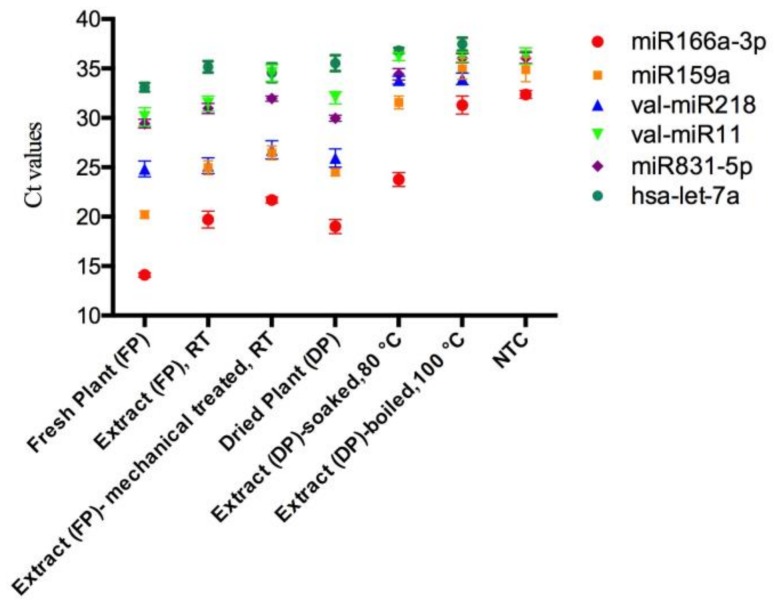
The raw Ct values of miRNAs in mistletoe plants and lyophilized mistletoe extracts detected by qRT-PCR. The Ct values of val-miR218 and hsa-let-7a in the NTCs were undetectable (Ct > 39). Sampling and analysis were carried out three independent times. The data regarding mistletoe extracts were extrapolated from dry weight. NTC represents no-template controls. RT represents room temperature.

To investigate the stability of mistletoe miRNAs during the herbal preparation process, several high expressed miRNAs including miR166a-3p, miR159a, miR831-5p, val-miR218 and val-miR11 were quantified in various mistletoe preparations by qRT-PCR. To determine the specificity of each primer set, no template controls (NTCs) for each miRNA were assessed simultaneously. The miRNAs were consistently and efficiently amplified in mistletoe plants and the majority of extracts. However, in the boiled extract, Ct values of miR166a-3p, miR159a, and miR831-5p cannot be distinguished from those of the corresponding NTCs (Figure 2). The Ct values of val-miR11 in the boiled extract, extract prepared as a tea, and the NTC cannot be distinguished with each other as well, suggesting that these miRNAs in corresponding extracts cannot be quantified by qRT-PCR.

To obtain the amount of plant miRNAs in different mistletoe preparations, each synthetic single-stranded miRNA was serially diluted and assessed using qRT-PCR to generate a standard curve. For each miRNA, the linear range of the Ct value and the dynamic quantification range of the expression level were listed in Table 2. As negative controls, the Ct values of human miRNA hsa-let-7a in the mistletoe plants and extracts were outside the linear range, confirming the specificity of the stem-loop qRT-PCR. The levels of each miRNA in mistletoe plants and extracts were calculated based on the reference to their corresponding standard curve (Supplementary Figure S1).

In agreement with our previous results [6], among tested miRNAs, miR166a-3p was the most abundant miRNA in fresh plants, followed by miR159a, val-miR218, val-miR11, and miR831-5p, respectively (Table 2). The miRNA profiles of dried plants and mistletoe extracts were different from that of fresh plants. MiR159a showed the highest copy number in dried plants and it remained the top level in extracts made from fresh plants, while miR166a-3p, val-miR11 and mi831-5p showed relatively low levels.

As expected, the miRNAs showed the highest levels in fresh mistletoe plants, ranging from miR831-5p with copy number of 3.3 × 10^7^ per gram to miR166a-3p with copy number of 5.0 × 10^11^ per gram. A significant decrease of miRNAs was observed in the extracts made from fresh plants. Since 50 mL of aqueous extract made from 5 g of raw plant materials produced approximately 1 g of lyophilized extract, compared with fresh plants, 0.3% miR166a-3p, 0.6% miR159a, 15.9% val-miR218, 7.0% val-miR11 and 4.8% miR831-5p remained in the extracts. The reduced levels of miRNAs might be caused by the degradation of miRNAs, while the incomplete release of miRNAs from crushed plants to the extract should be also taken into account. Medicinal plants often treated with mechanical treatments such as magnetic stir and ultrasonic agitation to better dissolve their bioactive components into the solvents they processed. Unexpected, mechanical treatment led to a significant decrease of miRNA amount. Compared with the extract without mechanical treatment, the amount of miR166a-3p, miR159a, val-miR218 and miR831-5p reduced by 81.1%, 69.1%, 68.3% and 63.3%, respectively, indicating that mechanical treatment might not enhance the release of miRNAs into a solvent, but increase miRNA damage.

A relatively high number of tested miRNAs were found in prepackaged dried mistletoe plants, with copy numbers ranging from 1.9 × 10^7^ to 1.2 × 10^10^, indicating that these miRNAs could survive air-drying and storage process. The dried mistletoe was soaked in hot water for 10 min to mimic the tea preparation. Indeed, the miRNAs were released from the raw materials to the water, but only miR166a-3p and miR159a with relatively low levels of 4.2 × 10^8^ and 6.3 × 10^7^ copies per gram were determined in the tea preparation. The Ct values of tested miRNAs in mistletoe decoction (boiled in water for 30 min) were either undetectable or outside the linear range. Apart from exposing to RNase and incomplete release of miRNAs from raw materials, heat might induce decrease of miRNAs as well.

### 2.3. The Stability of Synthetic miRNAs

Plant miRNAs might be associated with other molecules such as lipids, proteins and polysaccharides or be loaded into miRNA carriers such as exosomes, microvesicles, and protecting the miRNAs from degradation. On the other hand, 2′-*O*-methylation in plant miRNAs might also enhance their stability. To investigate whether free miRNAs could survive herb processing and the impact of 2′-*O*-methylation on miRNA stabilities, hsa-let-7a and hsa-let-7a with 2′-*O*-methylation were synthesized. These miRNAs were added to mistletoe extracts and processed with same conditions. As shown in Table 3 the Ct values of hsa-let-7a and 2′-OMe hsa-let-7a increased from approximately 23 (controls) to 33–37 (processed together with mistletoe extracts) that were outside the linear range (20.9–32.7), indicating negligible levels of synthetic miRNAs remained in the extracts. Compared with val-miR218 that showed a comparable Ct values (24.84 ± 0.81) in the starting raw materials, synthetic miRNAs underwent more intensive degradation during herb processing, suggesting that miRNAs that are free from any plant-derived molecules were susceptible to degradation. The 2′-*O*-methylation in plant miRNAs might help, but not enough to resist degradation in harsh environments.

**Figure 3:**
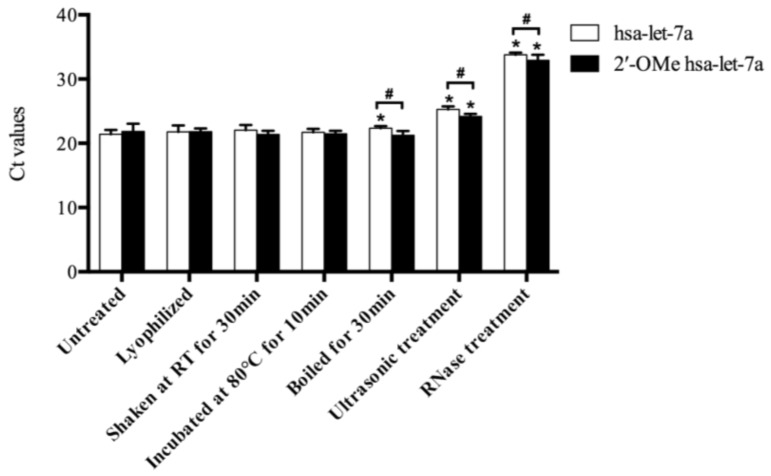
The Ct values of synthetic miRNAs with various treatments. Sampling and analysis were carried out three independent times. “*” represents a significant difference compared with untreated group (*p* < 0.05); “#” represents a significant difference between hsa-let-7a and 2′-OMe hsa-let-7a group (*p* < 0.05).

During the herb processing, drying, mechanical treatment, heat, and RNase that released from damaged plant cells might lead to the miRNA degradation. To explain the possible causes for miRNA degradation, synthetic miRNAs were treated with aforementioned conditions. As shown in Figure 3, comparable Ct values of both hsa-let-7a and 2′-OMe hsa-let-7a were found after lyophilization, intensively shaking and incubation at 80 °C, indicating simple drying, frequent movement and a certain degree of heat (less than 80 °C) might not be able to cause miRNA damage. After incubation at 100 °C for 30 min, a significant decrease (50%; 1-1/2^22.4–21.4^) of hsa-let-7a was observed, while 2′-OMe hsa-let-7a remained stable, indicating that methylation of miRNAs could enhance their heat stabilities. Ultrasonic treatment induced intensive degradation of miRNAs, and a significant reduction of both hsa-let-7a (93%; 1-1/2^25.3–21.4^) and 2′-OMe hsa-let-7a (80%; 1-1/2^24.2–21.8^) were detected after ultrasonic treatment. Moreover, almost complete damage of miRNAs was observed after RNase treatment. More than 99% of hsa-let-7a (99.98%; 1-1/2^33.8–21.4^) and 2′-OMe hsa-let-7a (99.95%; 1-1/2^32.9–21.8^) were digested by RNase. These results indicated that being exposed to RNase might cause a large extent degradation of plant miRNAs, while intensive mechanical treatment such as ultrasonic treatment might also induce severe damage of plant miRNA. On the other hand, methylation of plant miRNAs could enhance their resistance against heat, ultrasonic agitation and RNase digestion, but were not strong enough to protect miRNAs against the whole extreme environment.

## 3. Discussion

Plants have been used for medicinal purposes long before recorded history. However, the bioactive ingredients and their regulatory function remain to be discovered. In recent years, plant miRNAs as new bioactive constituents have attracted great attention. Rice miRNAs could enter the mammalian bloodstream and play a functional role in human metabolism [1]. The MIR2911 from honeysuckle was found to target influenza viruses and protect mice from influenza infections [2]. Plant derived miR159 significantly suppressed breast cancer cell proliferation by targeting transcription factor 7 (TCF7) [3]. Oral application of a cocktail that consisted of plant-based tumor suppressor miRNAs suppressed tumor burdens in mice [8]. It is well known that medicinal plants usually undergo extensive treatment such as grinding, drying, storage, soaking or boiling process before they are digested in vivo. As the potential bioactive ingredients, the stability of miRNAs during herb preparations is the precondition allowing them to function in vivo. Our previous study indicated that mistletoe miRNAs might be associated with its pharmacological effects such as anti-tumor, cardiovascular protective and neurological protective effects [6]. In this study, we tested the stability of mistletoe miRNAs in different mistletoe preparations.

As a frequently used adjuvant in cancer therapy, *V. album* extracts are commercially available in Europe [9], such as Isorel, abnobaVISCUM and Helixor. The majority of these commercial extracts use fresh or well-preserved (stored in liquid nitrogen) mistletoe as starting materials. As indicated by some companies (e.g., abnobaVISCUM), ascorbate phosphate buffer may ensure high yield of main ingredients (mistletoe lectins, viscotoxins). Accordingly, we prepared two extracts from fresh mistletoe plant with ascorbate phosphate buffer as the extract solution.

All the tested mistletoe miRNAs were found abundance in the fresh extracts (Table 2), even the amount reduced after ultrasonic treatment, some mistletoe miRNAs at levels of femtomolar per gram remained in the extracts. Commercial mistletoe extracts are often applied by intra-tumoral injection, or less often, subcutaneous injection or intravenous infusion [10], which display high bioavailability and facilitate rapid absorption. Injection of mistletoe extracts two to three times a week (20 mg per time) might introduce miR166a-3p (0.25 fmol; 7.4 × 10^9^ × 0.02 copies; for each injection), miR159a (0.37 fmol; 1.1 × 10^10^ × 0.02 copies; for each injection) and val-miR218 (0.25 fmol; 6.3 × 10^9^ × 0.02 copies; for each injection) at levels of attomolar or femtomolar into the tumor tissue or circulating system. It was reported that plant miRNAs could exert regulatory effects in animals at plasma concentration of fM [1,3]. Therefore, biologically relevant mistletoe miRNAs might probably be achieved via injection of commercial mistletoe extracts. Further animal experimental studies and even clinical data are needed to confirm the possibility of biologically relevant of plant miRNAs in mammals.

Dried mistletoe is widely available as a therapeutic herbal tea in continental Europe. Our results suggested that some mistletoe miRNAs (e.g., miR166a-3p and miR159a) were presented in mistletoe tea. Theoretically, 250 mL of hot mistletoe tea prepared from 5 grams of chopped mistletoe leaves may contain (a liquid mistletoe extract made from 5 g of dried mistletoe leaves could roughly create 1 g of lyophilized extract) 0.7 fmol (4.2 × 10^8^ copies) of miR166a-3p and 0.1 fmol (6.3 × 10^7^ copies) of miR159a. The bioavailability of these miRNAs after oral application remains to be elicited. A previous study indicated that plasma MIR2911 reached to a peak concentration of 1189 fM at 6 h after single gavage feeding of mice by honeysuckle decoction that contained 60 fmol (3.6 × 10^10^ copies) of MIR2911 [2]. The miR168a was presented in mouse liver and plasma after three days feeding with chow diet containing about 100 pmol (6.02 × 10^13^ copies) of miR168a [1]. Liang et al. described that the plant miRNAs including miR156a, miR162a, miR168a, miR172a, miR390a and miR528 were detected at fM levels in healthy human plasma after consumption of fruit juice with miRNA absorption rates of 0.04% to 1.31% [11], indicating miRNAs might have low bioavailability after oral application. Therefore, single consumption of mistletoe tea might not lead to the detectable plant miRNAs in human plasma. However, low bioavailability of plant miRNAs after oral application could not deny their bioactive potential. It has been indicated that miRNAs can act as hormones and execute their functions at several hundred copies per cell [1,12,13]. The bioavailability and therapeutic potential of mistletoe miRNAs through oral application remain to be validated.

It is noteworthy that, during the herbal preparation process, the degree of miRNA degradation varied among different miRNAs. For example, after mechanical treatment, the miR159a, val-miR218 and miR831-5p decreased by 69.1%, 68.3% and 63.3%, respectively, in the extract made from fresh plant, while 81.1% miR166a-3p was degraded (Table 2), indicating that miR166a-3p might be more sensitive to degradation. Meanwhile, even miR166a-3p showed a higher amount than miR159a in fresh mistletoe plant, and miR159a was found to have the largest copy number in various mistletoe preparations and extracts, indicating that they might be partnered with different molecules, which have different abilities to protect their integrity.

In addition, our results suggested that miRNAs degradation might attribute to the total RNA degradation during the herb preparing process. Similarly, the mice miRNAs (e.g., miR-122) were found degraded in total RNAs with low integrity, which were isolated from long-term stored frozen liver tissues [14]. In contrast, even RNA lost integrity in processed, cooked and in vitro digested soybean samples, and much higher levels of miRNAs (e.g., miR166, miR167 and miR168) were detected in the processed soybeans compared with the raw, uncooked beans [5]. It appeared that whether total RNA degradation correlated with miRNA degradation varied among different tissues and species, the matrix that miRNAs associated with might play a significant role, such as plant seeds that are rich in proteins and fat might offer more effective protection of miRNAs during plant processing.

Previous studies have explained the robustness of plant miRNAs. The 2′-*O*-methylated at their 3′ ends enhances their stability by protecting them from enzymatic digestion [15]. Plants are usually rich in secondary metabolites that may disrupt RNase activity and protect plant miRNAs from enzymatic environments [16,17,18]. Plant miRNAs are associated with RNA-binding proteins such as Argonaute proteins (AGOs) or encapsulated in nanoparticles such as exosomes and microvesicles, which might protect miRNAs from degradation [19,20,21]. In this study, methylated hsa-let-7a cannot survive enzymatic environments (e.g., RNase treatment and various mistletoe extracts), indicating that methylation of plant miRNAs might not be the key factor that keeps them stable during herb preparations. Furthermore, synthetic miRNAs degraded rapidly when they processed together with the mistletoe preparations (Table 3), indicating that the secondary metabolites in the extracts could not efficiently protect the miRNAs from degradation.

## 4. Materials and Methods

### 4.1. Plant Materials

One-year old *V. album* leaves and stems were collected at Berlin Botanical Garden in July 2017. The fresh mistletoe plants were snapped in liquid nitrogen and stored at −80 °C until use. Prepacked dried *V. album* leaves and stems of regular consumer standard were purchased from Alfred Galke GmbH, Bad Grund, Germany. They were collected in January 2015, air-dried, and stored at room temperature.

### 4.2. Preparations of Mistletoe Extracts

To mimic clinical use of mistletoe extracts, *V. album* fresh leaves and stems were grinded in liquid nitrogen. Five grams of powdered fresh mistletoe were added to 50 mL of extraction buffer (19.30 mM of Ascorbic acid, 1.31 mM of NaH_2_PO_4_·H_2_O, 11.34 mM of Na_2_HPO_4_·2H_2_O, and 17.35 mM of Na_3_PO4·12H_2_O, adjust pH of 7.5), mixed well, and centrifuged at 10,000× *g* for 20 min. The supernatant was then passed through 0.22 μm syringe filter and lyophilized. To evaluate the impact of mechanical damage during herb preparations, the powdered fresh mistletoe plant was added to an extraction buffer, treated with magnetic stir for 30 min, and then followed by ultrasonic treatment for a total of 30 min (10 min × 3 times). The resultant extract was centrifuged, filtered and lyophilized.

The prepacked dried *V. album* leaves and stems were ground to a fine powder using a homogenizer at room temperature. To mimic the typical European use of mistletoe as tea, 5 g of powdered plant materials were soaked in 50 mL of hot water (approximate 80 °C) for 10 min. To mimic the traditional Chinese preparations of herbs, 5 g of powdered mistletoe was boiled in 50 mL of RNase-free water (approximate 100 °C) for 30 min. These extracts were then centrifuged at 10,000× *g* for 20 min, and the supernatants were lyophilized and used for RNA isolation.

### 4.3. RNA Isolation

The fresh mistletoe leaves and stems were finely powdered by grinding in liquid nitrogen. The dried powdered plants and lyophilized extracts were weighed and processed for RNA isolation. Total RNA was extracted using RNA-Bee RNA isolation reagent (Amsbio, Abingdon, UK) according to the manufacturer’s protocol with slight modifications. Briefly, 100 mg of plant samples were mixed with 1 mL of RNA-Bee thoroughly, 0.2 mL of chloroform was added to the mixture, and centrifuged at 12,000× *g* for 20 min. The mixture was extract twice with chloroform, and the RNA was precipitated with half volume high salt solution (0.8 M sodium citrate, 1.2 M NaCl) and half volume isopropyl alcohol [22]. After washing twice with 75% ethanol, the RNA precipitate was dissolved in RNase-free water. Genomic DNA traces were eliminated using DNase I (Promega, Madison, WI, USA).

The quantity and quality of the RNA obtained were assessed spectrophotometrically at 230, 260 and 280 nm (Eppendorf, Hamburg, Germany). The A260/280 ratio was used to detect the contamination with DNA and proteins, while the A260/230 ratio was used to check for carbohydrate or phenol contamination. Quality and integrity of isolated RNA were verified by electrophoresis in 1.2% (*w*/*v*) agarose gel.

### 4.4. qRT-PCR

Five mistletoe miRNAs were selected and validated by qRT-PCR as previously described by Chen et al. [12]. The stem-loop primers for reverse transcription and primers for PCR were listed in Table 4. First-strand cDNA synthesis was performed using a TaqMan MicroRNA Reverse Transcription Kit (Thermo Scientific, Waltham, MA, USA) according to the manufacturer’s protocol. The reaction was carried out at 16 °C for 30 min, at 42 °C for 10 min, followed by heat-inactivation at 85 °C for 5 min.

Quantitative real-time PCR was conducted using the PowerUp™ SYBR^®^ Green Master Mix (Thermo Scientific) and PikoReal Real-Time PCR System (Thermo Scientific). The reactions were carried out with the following amplification conditions: activation at 50 °C for 2 min, 95 °C for 2 min, followed by 40 cycles of denaturation at 95 °C for 15 s, annealing at 55 °C for 15 s, and extension then at 72 °C for 30 s. All reactions were performed in three independent biological samples with three technical repeats. The melting curve was generated to test the specificity of PCR products and avoid the false-positive peaks. NTCs were included in all reactions. The Ct values were converted into absolute copy numbers using a standard curve that was independently generated with known quantities of the corresponding synthetic miRNA oligonucleotides (Metabion International AG, Steinkirchen, Germany).

### 4.5. Treatment of Synthetic miRNAs

The miRNA hsa-let-7a and hsa-let-7a with 2-*O*-methylation at the 3′ end nucleotide, which mimicked the chemistry of plant-derived miRNAs, were synthesized by Metabion International AG (Steinkirchen, Germany).

To investigate the possible factors that might cause miRNAs degradation, 50 µL of synthetic miRNA (100 pM) were treated with the following conditions: lyophilized and re-dissolved in 50 µL of RNase-free water; shook at room temperature by an orbital shaker incubator (Thermo Scientific) for 30 min; incubated in ultrasonic bath at room temperature for 10 min × 3 times; incubated at 80 °C in water bath for 10 min; and boiled at 100 °C for 30 min. For RNase treatment, 8 µL of synthetic miRNAs (125 pM) was incubated with 10 U of RNase If (New England Biolabs, Ipswich, MA, USA) in 1× Buffer in a total reaction volume of 10 µL at 37 °C for 30 min. The RNase was then inactivated by incubating at 70 °C for 20 min. After treatment, the synthetic miRNAs were cooled down on ice, spun down, and used for cDNA synthesis and qRT-PCR.

To investigate if synthetic miRNAs could survive the simulated conditions of herb processing, synthetic miRNAs were dissolved in RNase-free water, and processed together with mistletoe plants. Specifically, 25 µL of synthetic miRNAs (100 pM) was added to 1 mL of extracts made from fresh plants, then treated with or without mechanical treatment (including magnetic stir and ultrasonic treatment); or soaked in 1 mL of hot water (approximate 80 °C) containing dried powdered mistletoe for 10 min; or boiled with 1 mL boiling water containing dried powdered mistletoe for 30 min. The extracts were then centrifuged and lyophilized. The RNAs were isolated and dissolved in 25 µL of RNase-free water, and used for miRNA quantification.

### 4.6. Statistical Analysis

All data represent mean ± SD. Two-tailed Student’s *t*-tests were used to assess statistical significance. Values of *p* < 0.05 were considered significant.

## 5. Conclusions

This study evaluated the stability of mistletoe miRNAs during the herb preparation process. Even mistletoe miRNAs underwent intensive degradation during drying, storage, and extraction procedures, and the miRNAs with a large copy number were detected in the majority of mistletoe preparations. Injection of commercial mistletoe extracts made from fresh plants may facilitate a biologically relevant level of mistletoe miRNAs in patients’ tissues or circulating systems. However, single consumption of mistletoe tea made from dried chopped leaves might not introduce detectable miRNAs in human plasma.

The synthetic miRNAs cannot survive herb extraction procedures. Compared with heat and mechanical treatment, exposure to enzymatic environment might be the main cause of miRNA degradation. The methylation of plant miRNAs might enhance their ability against degradation, while being associated with other plant molecules could be a more efficient way for miRNA protection.

## Figures and Tables

**Table 1:** Comparison of RNA isolated from mistletoe plants and lyophilized mistletoe extracts.

RNA Parameters	Fresh Plant (FP)	Extract (FP), RT	Extract (FP)-Mechanical Treatment, RT	Dried Plant (DP)	Extract (DP)-Soaked, 80 °C	Extract (DP)-Boiled, 100 °C
RNA yield (µg/g)	630.3 ± 73.5	1404.5 ± 531.2	1236.9 ± 482.8	398.2 ± 109.3	931.8 ± 212.7	26.9 ± 7.3
A_260_/A_280_	2.10 ± 0.03	2.04 ± 0.05	2.00 ± 0.07	2.02 ± 0.04	1.97 ± 0.03	1.67 ± 0.11
A_260_/A_230_	2.31 ± 0.16	2.44 ± 0.09	2.54 ± 0.09	2.30 ± 0.33	2.18 ± 0.15	0.80 ± 0.25

Sampling and analysis were carried out three independent times. The data regarding mistletoe extracts were extrapolated from dry weight.

**Table 2:** The absolute quantification of miRNAs in mistletoe plants and lyophilized mistletoe extracts.

Plant miRNAs	Linear Range of Ct Values	Dynamic Quantification Range of miRNA Levels	Copy Number of miRNAs per Gram of Mistletoe Plants and Lyophilized Extracts					
			Fresh Plant (FP)	Extract (FP), RT	Extract (FP)-Mechanical Treatment, RT	Dried Plant (DP)	Extract (DP)-Soaked, 80 °C	Extract (DP)-Boiled, 100 °C
miR166a-3p	13.1–28.5	1 nM–10 fM	5.0 × 10^11^ ± 8.3 × 10^10^	7.4 × 10^9^ ± 4.6×10^9^	1.4 × 10^9^ ± 3.0 × 10^8^	1.2 × 10^10^ ± 6.1 × 10^9^	4.2 × 10^8^ ± 2.4 × 10^8^	NC
miR159a	17.8–32.9	1 nM–10 fM	3.9 × 10^11^ ± 1.2 × 10^11^	1.1 × 10^10^ ± 5.3 × 10^9^	3.4 × 10^9^ ± 2.0 × 10^9^	1.4 × 10^10^ ± 3.3 × 10^9^	6.3 × 10^7^ ± 3.0 × 10^7^	NC
val-miR218	15.4–33.0	1 nM–10 fM	7.9 × 10^9^ ± 3.2 × 10^9^	6.3 × 10^9^ ± 3.3 × 10^9^	2.0 × 10^9^ ± 1.5 × 10^9^	3.6 × 10^9^ ± 1.8 × 10^9^	NC	NC
val-miR11	20.7–32.6	100 pM–10 fM	1.2 × 10^8^ ± 6.4 × 10^7^	4.2 × 10^7^ ± 2.9 × 10^7^	NC	2.4 × 10^7^ ± 1.2 × 10^7^	NC	NC
miR831-5p	15.6–32.7	1 nM–1 fM	3.3 × 10^7^ ± 1.1 × 10^7^	7.9 × 10^6^ ± 3.3 × 10^6^	2.9 × 10^6^ ± 7.2 × 10^5^	1.9 × 10^7^ ± 6.1 × 10^6^	NC	NC
has-let-7a	20.9–32.7	100 pM–10 fM	NC	NC	NC	NC	NC	NC

Analyses were carried out three independent times. The data regarding mistletoe extracts were extrapolated from dry weight. The miRNA copies were extrapolated from dry weight. NC represents not calculated (the Ct values were outside the linear range). RT represents room temperature.

**Table 3:** The Ct values of synthetic miRNAs processed together with mistletoe preparations.

Synthetic miRNAs	Control	Mistletoe Extracts Containing Synthetic miRNAs			
		Extract (FP), RT	Extract (FP)-Mechanical Treatment, RT	Extract (DP)-Soaked, 80 °C	Extract (DP)-Boiled, 100 °C
hsa-let-7a	23.34 ± 0.47	34.81 ± 0.32	35.78 ± 0.79	35.63 ± 0.35	37.71 ± 0.58
2′-OMe hsa-let-7a	23.08 ± 0.43	33.09 ± 0.24	35.30 ± 0.30	35.28 ± 0.70	37.14 ± 0.69

Sampling and analysis were carried out three independent times.

**Table 4:** Primers for stem-loop qRT-PCT.

miRNAs	Sequence	RT Primer	Primer
miR166a-3p	UCGGACCAGGCUUCAUUCCCC	GTCGTATCCAGTGCGTGTCGTGGAGTCGGC AATTGCACTGGATACGACGGGGAA	F: AGTCGGACCAGGCTTCA R: CAGTGCGTGTCGTGGAG
miR159a	UUUGGAUUGAAGGGAGCUCU	GTCGTATCCAGTGCGTGTCGTGGAGTCGGC AATTGCACTGGATACGACAGAGCT	F: GCCGTTTGGATTGAAGG R: CAGTGCGTGTCGTGGAG
miR831-5p	AGGAAGACUGUAGAAGAGAUGAGG	GTCGTATCCAGTGCGTGTCGTGGAGTCGGC AATTGCACTGGATACGACCCTCAT	F: CTCAGGAAGACTGTAGAAGA R: CAGTGCGTGTCGTGGAG
val-miR218	GAUGAUCGCCACGUCGGAGGA	GTCGTATCCAGTGCAGGGTCCGAGGTATTC GCACTGGATACGACTCCTCC	F: AGGGATGATCGCCACG R: GTGCAGGGTCCGAGGT
val-miR11	CACUGUAGCACUUUUGACAAAG	GTCGT ATCCA GTGCA GGGTC CGAGG TATTC GCACT GGATA CGAC CTTTGT	F: GGGCACTGTAGCACTTTTG R: GTGCAGGGTCCGAGGT
hsa-let-7a	UGAGGUAGUAGGUUGUAUAGUU	GTCGTATCCAGTGCAGGGTCCGAGGTATTCGCACTGGATACGACAACTAT	F: GCCGTGAGGTAGTAGGTTGT R: GTGCAGGGTCCGAGGT

## Supplementary Materials

Supplementary materials are available online. Figure S1: Standard curves generated in qRT-PCR analysis.
