# Supplementary material for: The Stability of Medicinal Plant microRNAs in the Herb Preparation Process
Source: Molecules. 2018 Apr 16;23(4):919. doi: 10.3390/molecules23040919 (PMC6016954; doi:10.3390/molecules23040919)
Supplement: Supplementary file 1 [file molecules-23-00919-s001.pdf]

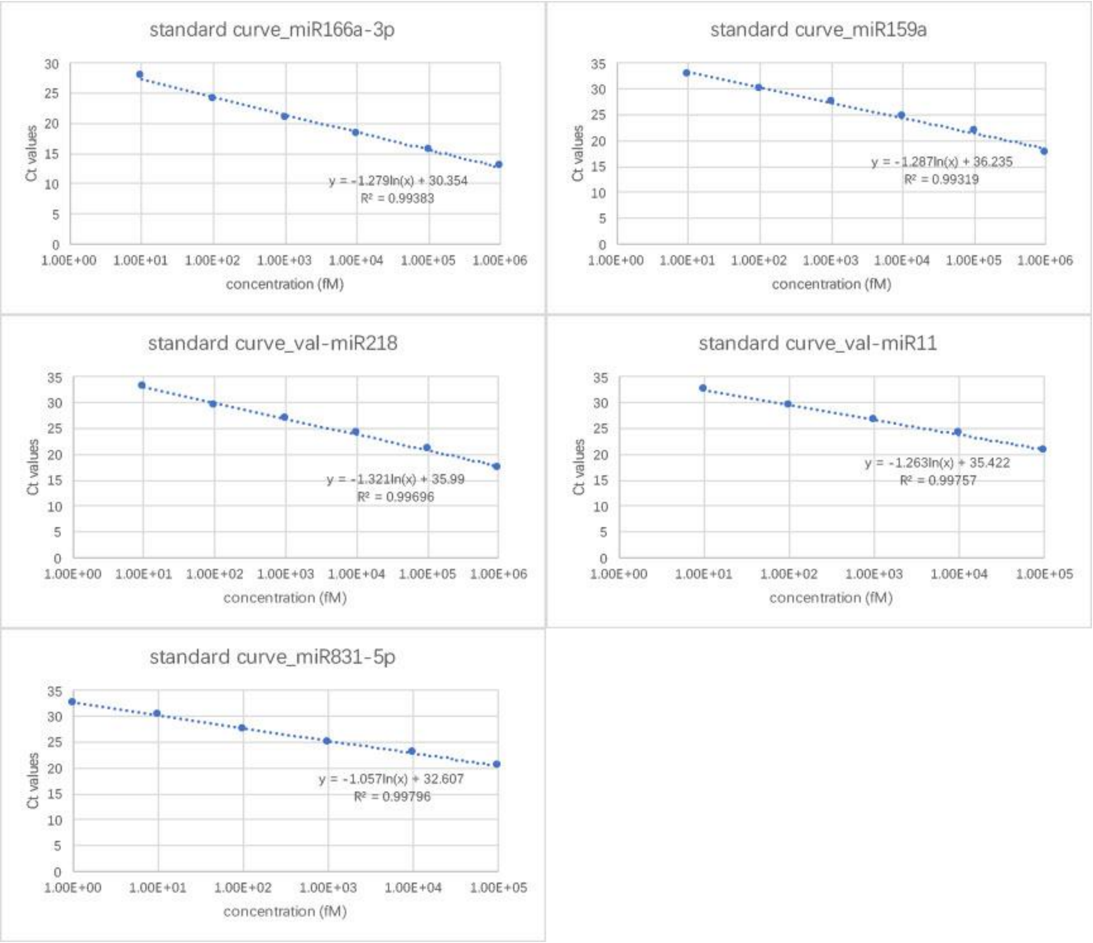

Supplementary Figure S1. Standard curves generated in qRT-PCR analysis. Synthetic single-stranded miRNA was serially diluted and assessed by the qRT-PCR assay. The resulting CT values were plotted against the miRNA amount to generate a standard curve.
